# Combined semirigid and flexible ureterorenoscopy for the treatment of large renal stones

**DOI:** 10.25122/jml-2023-0236

**Published:** 2023-09

**Authors:** Cosmin Cozma, Petrisor Geavlete, Razvan Multescu, Dragos Georgescu, Marius Bragaru, Bogdan Geavlete

**Affiliations:** 1 Carol Davila University of Medicine and Pharmacy, Bucharest, Romania; 2 Department of Urology, Sf. Ioan Emergency Clinical Hospital, Bucharest, Romania

**Keywords:** semirigid ureteroscopy, flexible ureteroscopy, large renal stones, renal lithiasis

## Abstract

Ureteroscopy is recognized as a minimally invasive and readily accessible method with low morbidity, favorable outcomes, and rapid post-interventional recovery. Recent advancements in rigid and flexible ureteroscopes have enhanced their efficiency, durability, and capability to accommodate accessory instruments. In this retrospective analysis, we evaluated 75 consecutive patients with large renal stones (stone burden between 2 and 4 cm) treated using a combination of semirigid and flexible ureteroscopy between January 1, 2020, and December 31, 2021. Stone properties and anatomical information were collected from the image archives derived from computed tomography (CT) and/or KUD radiography. Multiple ureteroscopy sessions were required for bigger stones. The length of the hospital stay, operation time, stone-free rate, preoperative and postoperative complications, and complication rates were examined. The average age of the patients was 52.7 years, with a mean stone burden of 31.45 mm. Most stones were in the renal pelvis, followed by the upper calyx. The average operative time was 56.2 minutes. After the initial ureteroscopy session, the stone clearance rate was 76%, which increased to 92% after two ureteroscopy sessions. Complication rates were evaluated using the Clavien-Dindo modified system, with an overall complication rate of 18.4%. Most of the complications were Clavien Grades I and II, with no grade V complication encountered. Large renal stones can be treated by combining semirigid and flexible ureteroscopy to reduce the operative time and protect the flexible ureteroscope.

## INTRODUCTION

The prevalence of urolithiasis, a significant health concern, is on the rise worldwide [1, 2]. When dealing with stones ranging from 1-2 cm in diameter, shockwave lithotripsy, and ureteroscopic fragmentation and retrieval are commonly employed methods. Ureteroscopy, in particular, offers higher stone-free rates and requires fewer treatments than shockwave lithotripsy [3, 4]. According to the 2022 European Association of Urology (EAU) recommendations, extracorporeal shockwave lithotripsy was recommended for treating renal calculi less than 2 cm in diameter, while percutaneous nephrolithotomy (PCNL) was suggested for treating renal calculi larger than 2 cm in diameter [5].

Advancements in techniques and technological instruments have expanded the options available to urologists for treating stone disease. A pivotal development in this subspecialty of surgery was the introduction of laser technology (Light Amplification by the Stimulated Emission of Radiation). Despite the advent of many other possibilities, the holmium: yttrium-aluminum-garnet (Ho: YAG) laser, referred to as the holmium laser in this article, has been the cornerstone laser platform for intracorporeal endoscopic stone lithotripsy for more than 20 years [6, 7]. Because of this, it is promoted as the gold standard among numerous international regulations [8].

Ureteroscopy is regarded as a minimally invasive, readily accessible method with low morbidity, which provides good results and rapid post-interventional recovery. Recent advancements in rigid and flexible ureteroscopes have enhanced their efficiency, durability, and capability to accommodate accessory instruments. Developing semi-rigid and flexible ureteroscopes with increasingly smaller dimensions has expanded the range of applications for endoscopic procedures in the urinary tract.

The primary objective of surgical treatment in managing upper urinary tract lithiasis is to achieve complete stone removal with the lowest possible morbidity rate. Currently, the therapeutic options for pyelo-calyceal lithiasis are represented by extracorporeal shock wave lithotripsy (ESWL), percutaneous nephrolithotomy (PCNL), the retrograde ureteroscopic approach (typically involving flexible ureteroscopy), and, rarely, open surgical treatment, usually secondary to complications. The selection of the therapeutic strategy is tailored to the unique characteristics of each patient.

## MATERIAL AND METHODS

We conducted a retrospective analysis of 75 consecutive patients with large renal stones (stone burden between 2 and 4 cm) treated with a combination of semirigid ureteroscopy and flexible ureteroscopy from January 1, 2020, to December 31, 2021, as detailed in Table 1. Stone characteristics and anatomical information were obtained from image archives of computed tomography (CT) and/or KUB radiography. Multiple ureteroscopy sessions were required for bigger stones. The greatest volume (length x width = mm^2^) was used to compute the stone surface area from the preoperative CT data. One month after the procedure, a follow-up evaluation was conducted utilizing non-contrast CT of the entire abdomen, a plain film of KUB for opaque stones, or ultrasonography for non-opaque stones.

**Table 1:** Demographic and clinical characteristics

Age (years)			52.7
**Gender**	**Male**		33
	**Female**		42
**Body weight (kg)**			84.56
**Mean stone burden (mm)**			31.45
**Stone location**		**Renal pelvis**	54
		**Upper pole**	21
		**Lower pole**	0
		**Middle pole**	0
**Pre-stented**			49

A successful surgical outcome was defined as the absence of remaining stone particles greater than 3 mm by the end of the surgical intervention. The surgical procedures, including flexible ureteroscopy (FURS) and semirigid ureteroscopy, were performed by a qualified urologist under the supervision of a senior urologist with substantial experience. The length of the hospital stay, operation time, stone-free rate, preoperative and postoperative problems, and complication rates were assessed.

### Surgical procedures

The surgical procedure began with a cystoscopy, a crucial step that precedes ureteroscopy. It is essential to evaluate the morphology and dimensions of the ureteral opening, as well as identify any potential associated bladder conditions. The next step involved placing a safety guidewire in the renal pelvis. This wire plays an important role in maintaining access for the ureteroscope in the upper urinary tract and facilitates its repeated passage. The guidewire also allows the ureteroscope to ascend into the urinary tract, helping to prevent parietal injuries during dilation procedures. The uretero-vesical junction and the intramural ureter represent the segments of the smallest caliber (approximately 3mm), which is why, if the caliber at the junction is too narrow, there is the possibility of using different dilation methods to allow the passage of the ureteroscope. Thus, before the existence of smaller ureteroscopes, ureteral dilation was a routine step in the operative protocol. Some authors demonstrated the absence of long-term sequelae after ureteral dilation with dilator balloons, such as Nottingham dilators or hydraulic dilation. However, modern instruments have reduced the need for dilation in approximately 14% of cases.

**Figure 1:**
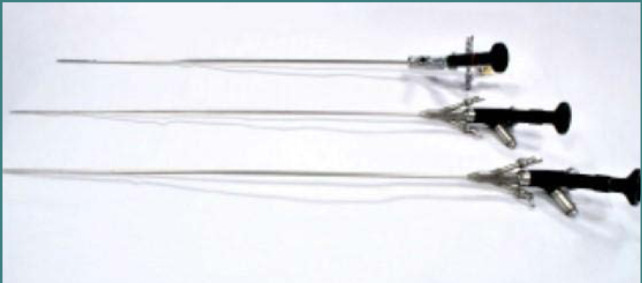
Different types of semirigid ureteroscopes

The progression of the semi-rigid ureteroscope begins under direct visual control, accompanied by the safety guidewire, and extends up to the level of the urinary bladder (Figure 1). Advancing the ureteroscope proximally is accomplished without applying excessive pressure, and it is essential to maintain the guidewire in the center of the endoscopic field, although some authors do not recommend using the safety guidewire normally. Extraction of calculi smaller than 6 mm can be done with a balloon catheter or forceps without requiring fragmentation. However, stones larger than 6 mm will be removed only after intracorporeal lithotripsy. As for the semi-rigid ureteroscopic approach to pyelo-calyceal stones, it can only be performed in certain situations. If the anatomy of the upper urinary tract does not allow this approach, the flexible ureteroscope will be used. lithotripsy techniques used in semi-rigid ureteroscopy are ballistic, ultrasonic, and laser lithotripsy. After removing intracorporeal lithotripsy and stone fragments, a flexible ureteroscope was introduced to verify the entire pyelo-caliceal system, ensuring that no fragments migrated to one of the poles during lithotripsy.

**Figure 2:**
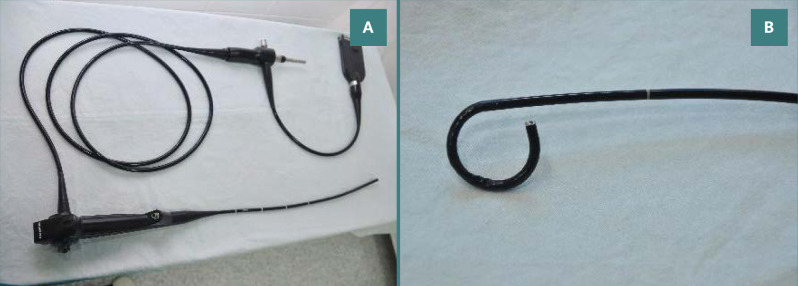
(A) A flexible ureteroscope and (B) its active deflection

During FURS, a small 3-mm diameter flexible endoscope, known as a ureteroscope, was introduced into the kidney through the urethra, bladder, and ureter to closely observe the kidney stone (Figures 2A-B). A flexible ureteroscope made by Olympus was used. The power source was a Dornier Medilas H Solvo 35 Holmium laser. After passing a laser fiber (often a 200 or 273 m holmium laser fiber) into the operating channel of the ureteroscope, the kidney stone was broken apart using laser energy. Larger fragments could be collected using a wire basket device passed through the working channel, while smaller fragments (less than 2 mm) could be allowed to pass naturally. After the procedure, patients usually eliminated any remaining stone particles in their urine over the week following treatment. This procedure typically required general anesthesia and an overnight hospital stay. In some cases, a temporary ureteral double-J stent was inserted at the end of the procedure to prevent ureter blockage caused by swelling of the lining cells.

### Data analysis

Data collected from medical records were processed and visualized using Microsoft Excel and Word, both part of Microsoft Office 18.2008.12711.0. Additionally, advanced statistical analysis was conducted using the Statistical Package for the Social Sciences (IBM SPSS) Version 26.

## RESULTS

Patient characteristics revealed a mean age of 52.7 years and an average stone burden of 31.45 mm. In 57 cases, solitary stones were present, while 18 cases involved multiple stones. For cases with multiple stones, the stone burden was calculated by summing the size of each stone. The most common stone location was in the renal pelvis, followed by the upper calyx. The average operative time was 56.2 minutes. In cases involving the upper pole stones, the procedure began with a semi-rigid ureteroscopy when the upper pole was accessible. If the upper pole was hard to access, we mobilized the stone in the renal pelvis and performed the lithotripsy there.

The stone-free rate (SFR) after the first ureteroscopy was 76%, and after two sessions of ureteroscopy, the SFR increased to 92% (Table 2).

**Table 2:** Perioperative characteristics

**Operative time (min)**	56.2	
**Fluoroscopy time (sec)**	20.1	
**Hospital stay (hours)**	58.4	
**Stone-free rate**	After one procedure	76%
	After two procedures	92%

Complication rates were evaluated using the Clavien-Dindo modified system. The overall complication rate was 18.4%. Most of the complications were Clavien Grade I and II. No grade V complication was encountered. The complications encountered are listed in Table 3.

**Table 3:** Complications of semirigid combined with flexible ureteroscopy

	Complications	Nr. Cases
Clavien I	Fever	3
	Mild hematuria	5
	Total	8
Clavien II	Urinary tract infections	4
Clavien IV	Sepsis requiring ICU management	1
Total		18.4%

## DISCUSSION

The semi-rigid ureteroscope is becoming increasingly popular, being less traumatic than the rigid one and providing superior results compared to the flexible ureteroscope in certain cases. It offers several advantages over the flexible ureteroscope, including lower costs and longer durability. Additionally, the larger dimensions of its working channel allow for a wider range of accessory instruments, ensuring improved visibility and irrigation during procedures. However, these advantages are counterbalanced by the difficulty of the semi-rigid ureteroscope to approach the proximal portion of the ureter and the pyelo-calyceal system. Due to the development of more compact, flexible, and semi-rigid ureteroscopes as well as reliable laser technology, the indications for ureteroscopic lithotripsy have expanded. There are several ways to do ureteroscopic lithotripsy, including laser, electrohydraulic, pneumatic, and ultrasound. Each technology has benefits and drawbacks.

**Figure 3:**
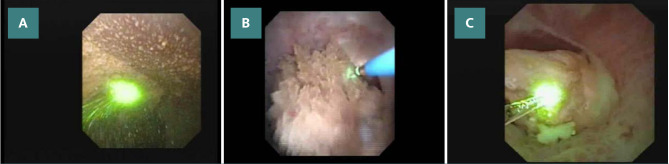
Different techniques of laser fragmentation. A: Dusting; B: Fragmenting; C: Popcorn

The Holmium laser, which replaced older lasers like the pulsed dye laser, has swiftly become the industry standard since its debut in urology practice in 1992 [9]. Unlike the pulsed dye laser and the neodymium: YAG laser, the Holmium laser operates on a photothermal effect rather than a photoacoustic effect for stone fragmentation [10]. Its development has been the result of ongoing research efforts. Today, a variety of hybrid strategies are available, including the ability to combine low energy (0.2–5 J) and high frequency (40–50 Hz) in contact mode, a technique known as “dusting”, in which the stone is broken down into tiny particles (submillimeter) that spontaneously evacuate [11]. Stone removal can be increased even further by switching to non-contact lithotripsy, sometimes known as the “pop-dusting” or “popcorn” method (Figure 3 A-C) [12]. Doing so removes the potential need for basket retrieval, which could reduce operational time and the need for a ureteric access sheath (UAS), which can lead to further complications [13].

In our study, the lithotripsy methods used were the Holmium laser lithotripsy as the main method, followed by the pneumatic one. The overall success rate in the present study was 92%. Comparatively, Atis *et al*. reported stone-free rates of 72% at postoperative day 1 and 76% at the 1-month follow-up for the semirigid ureteroscopy group, and 81.8% at postoperative day 1 and 86.4% at the 1-month follow-up for the flexible ureteroscopy group, although they did not combine both methods [14]. In a study involving 12 consecutive patients, 8 had total success, 3 had partial success, and 1 had residual fragments larger than 3 mm. Additionally, 88% of lower pole calyces were stone-free during the complication-free 3-month follow-up [15]. Miernik *et al*. reported a primary stone-free rate of 63.2% and an overall computed tomography (CT) controlled stone-free rate after 3 months (including staged procedures) of 81.8% [16].

In our study, intraoperative complications associated with semirigid ureteroscopy included stone migration in 21 cases, difficulty advancing the semirigid scope due to ureteral stenosis, and minor mucosal injuries. For cases of stone migration, we employed flexible ureteroscopy to access and manage the migrated fragment. In case of failure to advance the semirigid scope, we performed a double J ureteral stent insertion.

Regarding postoperative complications, our study primarily observed Clavien I and II complications, with one instance of a Clavien IV complication. The overall complication rate was 18.4%. In contrast, Mursi *et al*. found a 37% complication rate in a study that involved 90 patients treated with semirigid ureteroscopy [17]. Conversely, Miernik *et al*. observed Clavien II and III complications in only 7.9% of cases in a study combining semirigid and flexible ureteroscopy [18].

Some of the limitations of this study include a relatively small sample size, particularly in the younger subgroups, the absence of stone analysis, and the short duration of follow-up following semirigid ureteroscopy (sURS) for potential ureteric stricture or vesico-ureteric reflux development.

## CONCLUSION

Large renal stones can be treated by combining semirigid and flexible ureteroscopy to reduce the operative time and protect the flexible ureteroscope. Even if the entire lithiasis mass can be removed using the semirigid ureteroscope, verifying the entire pyelo-caliceal system using the flexible ureteroscope is mandatory to avoid missing a migrated stone fragment.
